# Supplementary material for: Molecular and structural basis of oligopeptide recognition by the Ami transporter system in pneumococci
Source: PLoS Pathog. 2024 Jun 5;20(6):e1011883. doi: 10.1371/journal.ppat.1011883 (PMC11192437; doi:10.1371/journal.ppat.1011883)
Supplement: S1 Table — (DOCX) [file ppat.1011883.s001.docx]

**S1 Table.** Percent Identity Matrix created by Clustal2.1 [1].

| **1 2 3 4 5** |
| --- |
| **1**: **AliC** 100.00 53.98 55.96 52.45 56.46 |
| **2**: **AmiA** 53.98 100.00 59.36 50.23 56.39 |
| **3**: **AliA** 55.96 59.36 100.00 51.53 59.08 |
| **4**: **AliD** 52.45 50.23 51.53 100.00 58.15 |
| **5**: **AliB** 56.46 56.39 59.08 58.15 100.00 |
