# Supplementary material for: Molecular and structural basis of oligopeptide recognition by the Ami transporter system in pneumococci
Source: PLoS Pathog. 2024 Jun 5;20(6):e1011883. doi: 10.1371/journal.ppat.1011883 (PMC11192437; doi:10.1371/journal.ppat.1011883)
Supplement: S2 Table — (DOCX) [file ppat.1011883.s002.docx]

| **Peptide name** | **Sequence** | **OBP** | **Origin determined by BLAST analysis** |
| --- | --- | --- | --- |
| **1** | FPPQSV | **AliD** | Ribosome-associated GTPase EngA (*Prevotella* species) |
| **2** | AIQSEKARKHN | **AliB** | 30S ribosomal protein S20 (multispecies) |
| **3** | PIVGGHEGAGV | **AliB** | NDMA-dependent alcohol dehydrogenase (multispecies) |
| **4** | VMVKGPGPGREST | **AliB** | 30S ribosomal protein S11 (multispecies) |
| **5** | AKTIKITQTR | **AmiA** | 50S ribosomal protein L30 (multispecies) |

**S2 Table. Chemically synthesized oligopeptides used in this work along with their corresponding Oligopeptide-binding protein (OBP).** Table adapted from Fauzy Nasher *et al*; 2018 [2] and expanded with data from Lucy J. Hathaway *et al*; 2014 [3].
