# Supplementary material for: Molecular and structural basis of oligopeptide recognition by the Ami transporter system in pneumococci
Source: PLoS Pathog. 2024 Jun 5;20(6):e1011883. doi: 10.1371/journal.ppat.1011883 (PMC11192437; doi:10.1371/journal.ppat.1011883)
Supplement: S3 Table — (DOCX) [file ppat.1011883.s003.docx]

**S3 Table.** Detailed composition for each substrate-binding pocket in AliD:peptide **1** complex

| **Pocket** | **P1** | | **P2** | | **P3** | **P4** | **P5** | **P6** |
| --- | --- | --- | --- | --- | --- | --- | --- | --- |
|  | |  |  |  | |  |  |  |
|  | Y50 | | Y50 | A52 | | A52 | S38 | F495 |
|  | A52 | | Y51 | T298 | | A56 | G39 | W300 |
| AliD pocket residues | K297 | | W498 | W300 | | N608 | D53 |  |
|  | V499 | | V499 | F481 | |  |  |  |
|  | I603 | | F518 | G497 | |  |  |  |
|  |  | | Y519 |  | |  |  |  |
| Aminoacid recognized | **F** | | **P** | **P** | | **Q** | **S** | **V** |
| Aminoacid preference | Hydrophobic | | Hydrophobic | Hydrophobic | | Hydrophobic | Polar | Hydrophobic |
