# Supplementary material for: Molecular and structural basis of oligopeptide recognition by the Ami transporter system in pneumococci
Source: PLoS Pathog. 2024 Jun 5;20(6):e1011883. doi: 10.1371/journal.ppat.1011883 (PMC11192437; doi:10.1371/journal.ppat.1011883)
Supplement: S4 Table — (DOCX) [file ppat.1011883.s004.docx]

**S4 Table.** Detailed composition for each substrate-binding pocket in AliB:peptide **2** complex.

| **Pocket** | **P1** | | **P2** | **P3** | **P4** | **P5** | **P6** | **P7** | **P8** | **P9** | **P10** | **P11** |
| --- | --- | --- | --- | --- | --- | --- | --- | --- | --- | --- | --- | --- |
|  | |  |  |  |  |  |  |  |  |  |  |  |
|  | A54 | | N52 | A54 | S41 | Y302 | S40 | S40 | E351 | F495 | Y37 | Y37 |
|  | S57 | | R53 | D58 | A55 | H497 | S41 | R583 | S353 | W300 | V38 | R273 |
| AliB pocket residues |  | | Y483 | T300 | Y483 |  | Y248 |  | Y349 |  | S40 | R583 |
|  |  | | F521 | Y302 | F484 |  | E440 |  |  |  | Y252 | T586 |
|  |  | | W500 | R583 | Y496 |  | F478 |  |  |  | L257 |  |
|  |  | |  |  |  |  | D479 |  |  |  |  |  |
| Aminoacid recognized | **A** | | **I** | **Q** | **S** | **E** | **K** | **A** | **R** | **K** | **H** | **N** |
| Aminoacid preference | Hydrophobic | | Hydrophobic | Polar | Polar | Polar | Polar | Hydrophobic | Polar | Hydrophobic | Hydrophobic | Hydrophobic |
|  |  | |  |  |  |  |  |  |  | Polar | Polar | Polar |
