# Supplementary material for: Molecular and structural basis of oligopeptide recognition by the Ami transporter system in pneumococci
Source: PLoS Pathog. 2024 Jun 5;20(6):e1011883. doi: 10.1371/journal.ppat.1011883 (PMC11192437; doi:10.1371/journal.ppat.1011883)
Supplement: S5 Table — (DOCX) [file ppat.1011883.s005.docx]

**S5 Table.** Detailed composition for each substrate-binding pocket in AliB:peptide **3** complex.

| **Pocket** | **P1** | **P2** | **P3** | **P4** | **P5** | **P6** | **P7** | **P8** | **P9** | **P10** | **P11** |
| --- | --- | --- | --- | --- | --- | --- | --- | --- | --- | --- | --- |
|  | A54 | N52 | A54 | S41 | S40 | S40 | F304 | Y302 | Y302 | R273 | Y37 |
|  | D503 | R52 | D58 | A55 | S41 | S41 | Y349 | Y349 | I362 | V445 | V38 |
| AliB pocket residues |  | Y483 | T300 | Y483 | R583 | Y252 | K437 | H497 | V581 | R583 | Y252 |
|  |  | F521 | Y302 | R583 |  | E440 | D443 |  | R583 |  | L260 |
|  |  | W500 | R583 |  |  | D443 | V450 |  |  |  | R273 |
|  |  |  |  |  |  |  | H497 |  |  |  | F275 |
| Aminoacid recognized | **P** | **I** | **V** | **G** | **G** | **H** | **E** | **G** | **A** | **G** | **V** |
| Aminoacid preference | Hydrophobic | Hydrophobic | Hydrophobic | Hydrophobic | Hydrophobic | Hydrophobic | Hydrophobic | Hydrophobic | Hydrophobic | Hydrophobic | Hydrophobic |
|  |  |  | Polar | Polar | Polar | Polar | Polar | Polar | Polar | Polar |  |
