# Supplementary material for: Molecular and structural basis of oligopeptide recognition by the Ami transporter system in pneumococci
Source: PLoS Pathog. 2024 Jun 5;20(6):e1011883. doi: 10.1371/journal.ppat.1011883 (PMC11192437; doi:10.1371/journal.ppat.1011883)
Supplement: S6 Table — (DOCX) [file ppat.1011883.s006.docx]

**Table S6.** Detailed composition for each substrate-binding pocket in AliB:peptide **4** complex.

| **Pocket** | **P1** | **P2** | **P3** | **P4** | **P5** | **P6** | **P7** | **P8** | **P9** | **P10** |
| --- | --- | --- | --- | --- | --- | --- | --- | --- | --- | --- |
|  | A54 | N52 | A54 | S41 | Y320 | Y302 | S40 | Y37 | Y37 | Y35 |
|  | S57 | R52 | D58 | A55 | F478 | Y349 | Y252 | V38 | V38 | A597 |
| AliB pocket residues | T604 | Y483 | T300 | F478 | R583 | I362 | V445 | Y252 | R583 | V607 |
|  |  | F521 | Y302 | D479 |  | H487 |  | L260 |  | Y610 |
|  |  | W500 | R583 | F484 |  | V581 |  | R273 |  | L613 |
|  |  |  |  |  |  |  |  | F275 |  |  |
| Aminoacid recognized | **V** | **M** | **V** | **K** | **G** | **P** | **G** | **P** | **G** | **R** |
| Aminoacid preference | Hydrophobic | Hydrophobic | Hydrophobic | Hydrophobic | Hydrophobic | Hydrophobic | Hydrophobic | Hydrophobic | Hydrophobic | Hydrophobic |
|  |  |  | Polar | Polar | Polar | Polar | Polar | Polar | Polar | Polar |
