# Supplementary material for: Molecular and structural basis of oligopeptide recognition by the Ami transporter system in pneumococci
Source: PLoS Pathog. 2024 Jun 5;20(6):e1011883. doi: 10.1371/journal.ppat.1011883 (PMC11192437; doi:10.1371/journal.ppat.1011883)
Supplement: S7 Table — (DOCX) [file ppat.1011883.s007.docx]

**S7 Table.** Detailed composition for each substrate-binding pocket in AmiA:peptide **5** complex.

| **Pocket** | **P1** | **P2** | **P3** | **P4** | **P5** | **P6** | **P7** | **P8** | **P9** | **P10** |
| --- | --- | --- | --- | --- | --- | --- | --- | --- | --- | --- |
|  | T59 | S54, K55, | N56 | A43 | A305, Y352, | D443 | T42 | R276 | Y39 | Y37 |
|  | Y187 | E134, Y486, | Y486 | L482 | Y449 | S446 | Y255 | A305 | V40 | Y39 |
| AmiA pocket residues | S610 | V502, W504, | S503 | Y486 | R452 | Y449 |  | I356 | T42 | Y274 |
|  |  | E524, T525, |  | Y487 | G500 | F481 |  | R452 | Y255 | D614 |
|  |  | K527, T528, |  | V502 | M582 | L482 |  | M586 | L263 | F617 |
|  |  | Y529 |  |  | A585 |  |  |  |  |  |
| Aminoacid recognized | **A** | **K** | **T** | **I** | **K** | **I** | **T** | **Q** | **T** | **R** |
| Aminoacid preference | Hydrophobic | Hydrophobic | Hydrophobic | Hydrophobic | Hydrophobic | Hydrophobic | Hydrophobic | Hydrophobic | Hydrophobic | Hydrophobic |
|  | Polar | Polar | Polar |  | Polar |  | Polar | Polar | Polar | Polar |
