# Supplementary material for: Molecular and structural basis of oligopeptide recognition by the Ami transporter system in pneumococci
Source: PLoS Pathog. 2024 Jun 5;20(6):e1011883. doi: 10.1371/journal.ppat.1011883 (PMC11192437; doi:10.1371/journal.ppat.1011883)
Supplement: S8 Table — (DOCX) [file ppat.1011883.s008.docx]

**S8 Table.** Strains and plasmids list

Strain or plasmid Serotype and relevant Genotype^a^ Resistance Source or Reference

***Streptococcus pneumoniae***

D39 2 None NCTC7466

TIGR4 4 None [4]

MNZ41 Non-typeable strain Tmp [5]

***Escherichia coli***

DH5α Δ*(lac)U169, endA1, gyrA46, hsdR17, Φ80*Δ*(lacZ)M15,* None Bethesda Research Labs,

*recA1, relA1, supE44, thi-1* Gaithersburg, U.S.

BL21(DE3) *E.coli* B, *F- dcm ompT hsdS gal λ*(DE3), T7 polymerase None Novagen, Merck KGaA,

gene under control of the lacUV5 promoter Darmstadt, Germany

B834(DE3) *E.coli* B, *F- dcm ompT hsdS gal λ*(DE3) Novagen, Merck KGaA,

Darmstadt, Germany

**Plasmids**

pET28 Protein expression vector Km^r^ Novagen

pTP1 pET28 expression vector with a TEV protease Km^r^, Erm^r^ [6]

cleavage site

p1077 pTP1 with TIGR4 *sp_1891* (*amiA*) for protein Km^r^  This work

production and mice immunization.

p1349 pTP1 with D39 *spd_0334* (*aliA*) for protein Km^r^ This work

production and mice immunization.

p1345 pTP1 with MNZ41 (*aliC*) for protein Km^r^ This work

production and mice immunization.

p1347 pTP1 with MNZ41 (*aliD*) for protein Km^r^ This work

production and mice immunization.

Tmp, trimethoprim; Km, kanamycin; Erm, Erythromycin and r, resistant
