# Supplementary material for: Molecular and structural basis of oligopeptide recognition by the Ami transporter system in pneumococci
Source: PLoS Pathog. 2024 Jun 5;20(6):e1011883. doi: 10.1371/journal.ppat.1011883 (PMC11192437; doi:10.1371/journal.ppat.1011883)
Supplement: S9 Table — (DOCX) [file ppat.1011883.s009.docx]

**S9 Table.** Primer list

Primer purpose Primer Sequence (5’-3’)

**Recombinant protein production**

*sp_1891* (TIGR4; *amiA*) AmiA_1562 5`-GCGCGCGCTAGCAGTTCTTCAAAATCATCTGATTC-3`

AmiA_1563 5`- GCGCGCGAGCTCTTACTTCACATGACTTGCCAATTC -3`

*spd_0334* (D39; *aliA*) AliA_1931 5`- AATTGCTAGCTCTGGATCAGGTTCAAGC -3`

AliA_1932 5`- GCGCGAGCTCATTTCACATGTTTTGC -3`

*aliC* (MNZ41; *aliC*) AliC_1921 5`- GCGCGCGCTAGCAAAAGTGAAAAGAATGC -3`

AliC_1922 5`- GCGCGCGAGCTCATTTTATGTGCTTTTC -3`

*aliD* (MNZ41; *aliD*) AliD_1923 5`- GCGCGCACTAGTTCAGATACAAAAACTTAC -3`

AliD_1924 5`- GCGCGCAAGCTTATTTAACATGTTTTTCTGC -3`

*spd_1357 (D39; aliB)* AliB_1288 5’ -GCGTGCTAGCGGAAATTCTAGCACTGCATC-3` AliB_1277 5’ -GCGCGAGCTCTTATTTGACATGTTTTGCC-3`

Restriction sites are underlined
