# Supplementary material for: Molecular and structural basis of oligopeptide recognition by the Ami transporter system in pneumococci
Source: PLoS Pathog. 2024 Jun 5;20(6):e1011883. doi: 10.1371/journal.ppat.1011883 (PMC11192437; doi:10.1371/journal.ppat.1011883)
Supplement: S10 Table — (DOCX) [file ppat.1011883.s010.docx]

**S10 Table.** Crystallographic data collection and refinement statistics*

|  | **AliD** | | **AliD:Peptide 1** | | **AliC** | **AliB:Unk Peptide** | **AliB:Peptide 2** | **AliB:Peptide 3** | **AliB:Peptide 4** | **AmiA:Unk Peptide** | **AmiA:Peptide 5** |
| --- | --- | --- | --- | --- | --- | --- | --- | --- | --- | --- | --- |
| **Data collection** | |  |  |  | |  |  |  |  |  |  |
| Wavelength (Å) | 0.97926 | | 0.97925 | 0.97928 | | 0.97926 | 0.97926 | 0.97926 | 0.97918 | 0.97924 | 0.97926 |
| Space group | P 2_1_ 2_1_ 2_1_ | | P 1 | C 1 2 1 | | P 1 2_1_ 1 | P 1 2_1_ 1 | P 1 2_1_ 1 | P 1 2_1_ 1 | P 2_1_ 2_1_ 2_1_ | P 1 2_1_ 1 |
| Unit cell *a, b, c* (Å) | 57.24 93.00 111.65 | | 52.3 67.3 109.8 | 185.88 60.43 63.60 | | 49.43 113.46 58.73 | 52.98, 111.50, 56.28 | 64.20 108.84 86.64 | 49.76, 114.14, 58.98 | 56.60 102.70 107.57 | 56.65, 206.76, 96.79 |
| Unit cell α,β,γ (º) | 90, 90, 90 | | 84.05 87.26 71.72 | 90.00 96.25 90.00 | | 90, 107.52, 90 | 90, 108.40, 90 | 90.00 100.05 90.00 | 90, 107.66, 90 | 90, 90, 90 | 90, 90.76, 90 |
| T (K) | 100 | | 100 | 100 | | 100 | 100 | 100 | 100 | 100 | 100 |
| X-ray source | Synchrotron | | Synchrotron | Synchrotron | | Synchrotron | Synchrotron | Synchrotron | Synchrotron | Synchrotron | Synchrotron |
| Resolution range (Å) | 48.75–(1.86–1.80) | | 46.63–(2.05–1.98) | 57.52–(2.46–2.38) | | 43.53–(1.70–1.65) | 48.24–(2.37–2.29) | 47.02–(1.71–1.66) | 47.41–(1.52–1.49) | 47.64–(1.55–1.50) | 48.61–(1.82–1.76) |
| Unique reflections | 64025 (3371) | | 94495 (4650) | 28215 (2820) | | 72522 (3496) | 27597 (2715) | 134554 (6595) | 99634 (4851) | 97923 (4649) | 213511 (10351) |
| Completeness (%) | 99.99 (99.99) | | 97.10 (96.02) | 98.90 (99.20) | | 98.10 (96.80) | 98.60 (98.40) | 97.60 (96.10) | 97.70 (95.80) | 97.20 (95.10) | 97.50 (95.90) |
| Multiplicity | 12.8 (13.4) | | 3.5 (3.6) | 5.2 (5.3) | | 4.8 (4.5) | 4.5 (4.6) | 6.8 (7.0) | 5.4 (5.5) | 7.6 (7.0) | 6.9 (6.5) |
| *R_merge_^a^* | 0.087 (1.089) | | 0.10 (0.82) | 0.080 (0.657) | | 0.089 (0.704) | 0.112 (0.695) | 0.075 (0.792) | 0.053 (0.671) | 0.068 (0.549) | 0.096 (0.768) |
| *R_pim_^b^* | 0.025 (0.305) | | 0.067 (0.513) | 0.037 (0.309) | | 0.044 (0.371) | 0.058 (0.359) | 0.031 (0.322) | 0.025 (0.312) | 0.026 (0.218) | 0.039 (0.3323) |
| *<I/σ(I)>* | 16.2 (2.2) | | 7.3 (1.5) | 11.2 (2.3) | | 10.1 (2.1) | 10.7 (2.4) | 13.9 (2.2) | 17.3 (2.7) | 18.9 (4.1) | 12.7 (2.3) |
| CC1/2 | 0.99 (0.85) | | 0.99 (0.78) | 0.99 (0.80) | | 0.99 (0.70) | 0.995 (0.74) | 0.99 (0.77) | 0.99 (0.78) | 0.99 (0.84) | 0.998 (0.79) |
| **Refinement** |  | |  |  | |  |  |  |  |  |  |
| Resolution range (Å) | 48.75–1.80 | | 46.63–2.1 | 57.52–2.38 | | 43.53–1.65 | 48.24–2.29 | 47.02–1.66 | 47.41–1.49 | 47.64–1.50 | 48.61–1.76 |
| *R_work_/R_free_^c^* | 0.1760/ 0.2113 | | 0.1906/ 0.2247 | 0.1765/ 0.2252 | | 0.1557/ 0.1903 | 0.1654/ 0.2192 | 0.1663/ 0.2050 | 0.1381/ 0.1749 | 0.1569/ 0.1786 | 0.1570/ 0.1976 |
| No. Atoms |  | |  |  | |  |  |  |  |  |  |
| Protein | 4972 | | 9894 | 4918 | | 4945 | 4946 | 9910 | 4936 | 4947 | 19831 |
| Water | 549 | | 598 | 128 | | 706 | 235 | 1258 | 802 | 673 | 2068 |
| Ligand | – | | 96 | – | | 46 | 90 | 140 | 76 | 51 | 324 |
| **R.m.s. deviations** |  | |  |  | |  |  |  |  |  |  |
| Bond length (Å) | 0.006 | | 0.004 | 0.005 | | 0.006 | 0.003 | 0.008 | 0.005 | 0.006 | 0.007 |
| Bond angles (°) | 0.90 | | 0.59 | 1.05 | | 0.78 | 0.62 | 0.88 | 0.72 | 0.88 | 0.89 |
| **Ramachandran**  Favored/outliers (%) | 97/0 | | 97/0 | 97/0 | | 97/0 | 98/0 | 97/0 | 98/0 | 98/0 | 98/0 |
| Monomers per AU | 1 | | 2 | 1 | | 1 | 1 | 2 | 1 | 1 | 4 |
| **Average B-factor**  Macromolecules | 32.39  31.85 | | 41.31  41.16 | 58.38  58.55 | | 23.06  21.33 | 32.74  32.34 | 29.30  27.86 | 21.42  19.18 | 17.50  15.43 | 27.41  26.02 |
| Ligands | – | | 48.18 | – | | 44.67 | 43.48 | 61.16 | 38.50 | 26.27 | 49.83 |
| Solvent | 37.32 | | 42.09 | 52.30 | | 35.25 | 36.92 | 37.08 | 35.38 | 32.04 | 37.16 |
| **PDB code** | 8QLC | | 8QLG | 8QLH | | 8QLJ | 8QLK | 8QLM | 8QLV | 8A42 | 8QM0 |

*Values between parentheses correspond to the highest resolution shells

^a^*R*_merge_ = Σ_hkl_ Σ_i_ | I_i_(hkl) – [I(hkl)] | / Σ_hkl_Σ_i_I_i_(hkl), where Σ_i_I_i_(hkl) is the *i*-th measurement of reflection hkl, [I(hkl)] is the weighted mean of all measurements.

^b^*R*_pim_ = Σ_hkl_[1/(N – 1)] ^1/2^ Σ_i_ | I_i_(hkl) – [I(hkl)] | / Σ_hkl_Σ_i_I_i_(hkl), where Σ_i_I_i_(hkl) is the *i*-th measurement of reflection hkl, [I(hkl)] is the weighted mean of all measurements and N is the redundancy for the hkl reflection.

^c^*R*_work_/*R*_free_ = Σ_hkl_| F_o_ - F_c_ | / Σ_hkl_ | F_o_ |, where F_c_ is the calculated and F_o_ is the observed structure factor amplitude of reflection hkl for the working / free (5%) set, respectively

For the calculation of average b factors of each independent chain (protein and peptide) we used *phenix.b_factor_statistics model.pdb* command
