# Supplementary material for: Molecular and structural basis of oligopeptide recognition by the Ami transporter system in pneumococci
Source: PLoS Pathog. 2024 Jun 5;20(6):e1011883. doi: 10.1371/journal.ppat.1011883 (PMC11192437; doi:10.1371/journal.ppat.1011883)
Supplement: S11 Table — (DOCX) [file ppat.1011883.s011.docx]

**S11 Table.** Nano LC-MS/MS data acquisition parameters.

| LC-Parameters | |
| --- | --- |
| Instrument | NanoAcquity UPLC (Waters GmbH, Eschborn, Germany) |
| Trap column | NanoAcquity UPLC 2G-V/M trap Symmetry C18 pre-column, 2 cm length, 180 µm ID and 5 µm particle size (Waters GmbH, Eschborn, Germany) |
| Analytical column | NanoAcquity BEH130 C18 column, 10 cm length, 100 µM ID and 1.7 µm particle size (Waters GmbH, Eschborn, Germany) |
| Buffer system | Binary buffer system consisting of buffer A (0.5% DMSO in water with 0.1% acetic acid) and buffer B (5% DMSO in acetonitrile with 0.1% acetic acid) |
| Flow rate | 400 nl/min |
| Gradient | 0 min 1% B, 2 min 5% B, 65 min 25% B, 90 min 60% B, 91 min 99% B |
| Column oven temperature | 40°C |
|  | |
| **MS-Parameters** | |
| Instrument | LTQ-Orbitrap Velos mass spectrometer (Thermo Fisher Scientific, Dreieich, Germany) |
| Ion Source | Nano-ESI source and installed with a Picotip Emmitter (New Objective, USA). |
| Operation mode | Data-dependent acquisition |
|  | |
| **Full MS-Parameters** | |
| MS scan resolution | 30,000 |
| AGC target | 1e6 |
| Max. ion injection time for the MS scan | 10ms |
| Scan range | 325 to 1525 m/z |
| Spectra data type | Profile |
|  | |
| **MS2-Parameters** | |
| MS/MS AGC target | 1e4 |
| Max. ion injection time for the MS/MS scans | 100 ms |
| Selection for MS/MS | 20 most abundant isotope patterns with charge ≥ 2 from the survey scan |
| Isolation width | 2 m/z |
| Dissociation mode | collision-induced dissociation (CID) |
| Normalized collision energy | 35% |
| Dynamic exclusion | 60 s |
| Spectra data type | centroid |
| Charge exclusion | Unassigned, 1, 4 and above |
