# Supplementary figures and images for: Molecular and structural basis of oligopeptide recognition by the Ami transporter system in pneumococci
Source: PLoS Pathog. 2024 Jun 5;20(6):e1011883. doi: 10.1371/journal.ppat.1011883 (PMC11192437; doi:10.1371/journal.ppat.1011883)

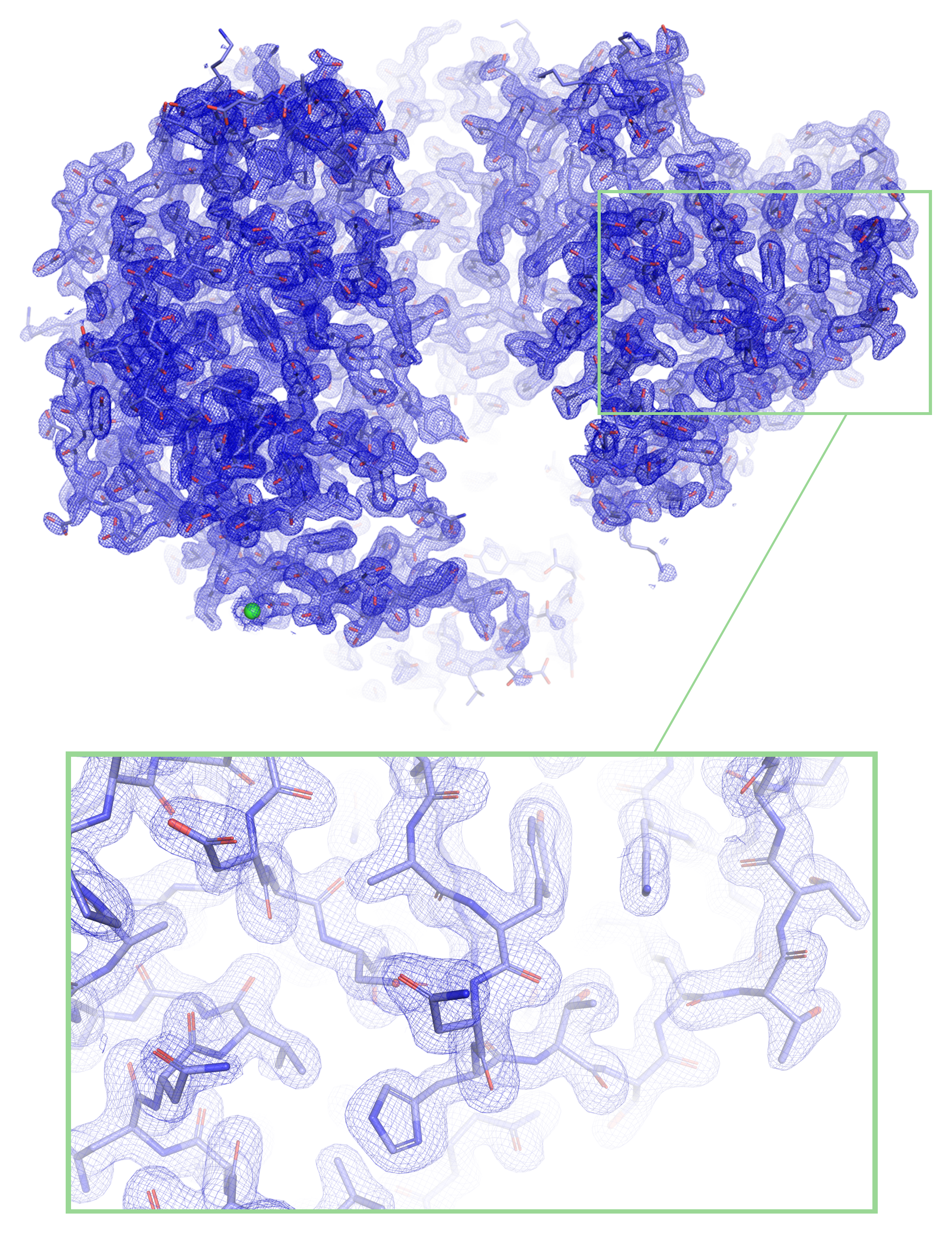

Supplement: S2 Fig — The protein structure is depicted in blue capped sticks. A Mg2+ atom, contributing to crystal packing, is represented as a green sphere. The lower panel provides a detailed view of the boxed area in the upper panel, highlighting electron densities at 1.8 Å resolution. (TIF) [file ppat.1011883.s013.tif]

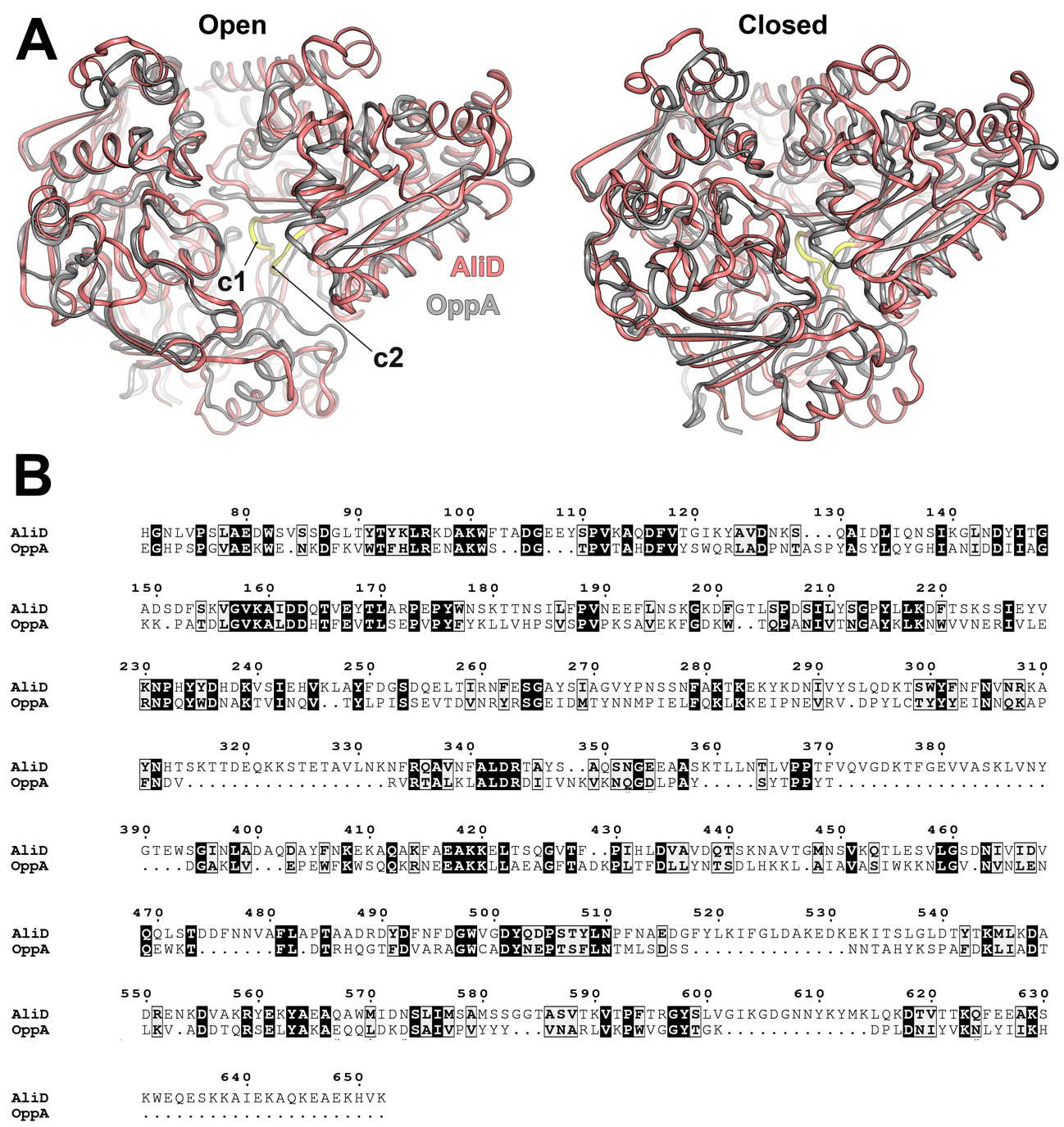

Supplement: S3 Fig — (A) Left panel; superposition of the structures of AliD from S. pneumoniae (this work, colored in red) and OppA from S. typhimurium [colored in gray, PDB 1RKM [25]], both in the open conformation (rmsd of 3.18 Å for 349 Cα atoms). Right panel; structure superposition between AliD from S. pneumoniae (this work, colored in red) and OppA from S. typhimurium [colored in gray, PDB 1B4Z [26]], both in the closed conformation (rmsd of 3.20 Å for 347 Cα atoms). Both proteins are represented as cartoon oval. The two crossover connecting regions in AliD are colored yellow and labeled as "c1" and "c2", respectively. (B) Sequence alignment of AliD from S. pneumoniae and OppA from S. typhimurium, generated by T-COFFEE [22] and drawn with ESPript [23]. Sequence identity, estimated with Clustal2.1, is 25,76%. Identities are boxed in black. Similarities are boxed in gray according to physicochemical properties. (TIF) [file ppat.1011883.s014.tif]

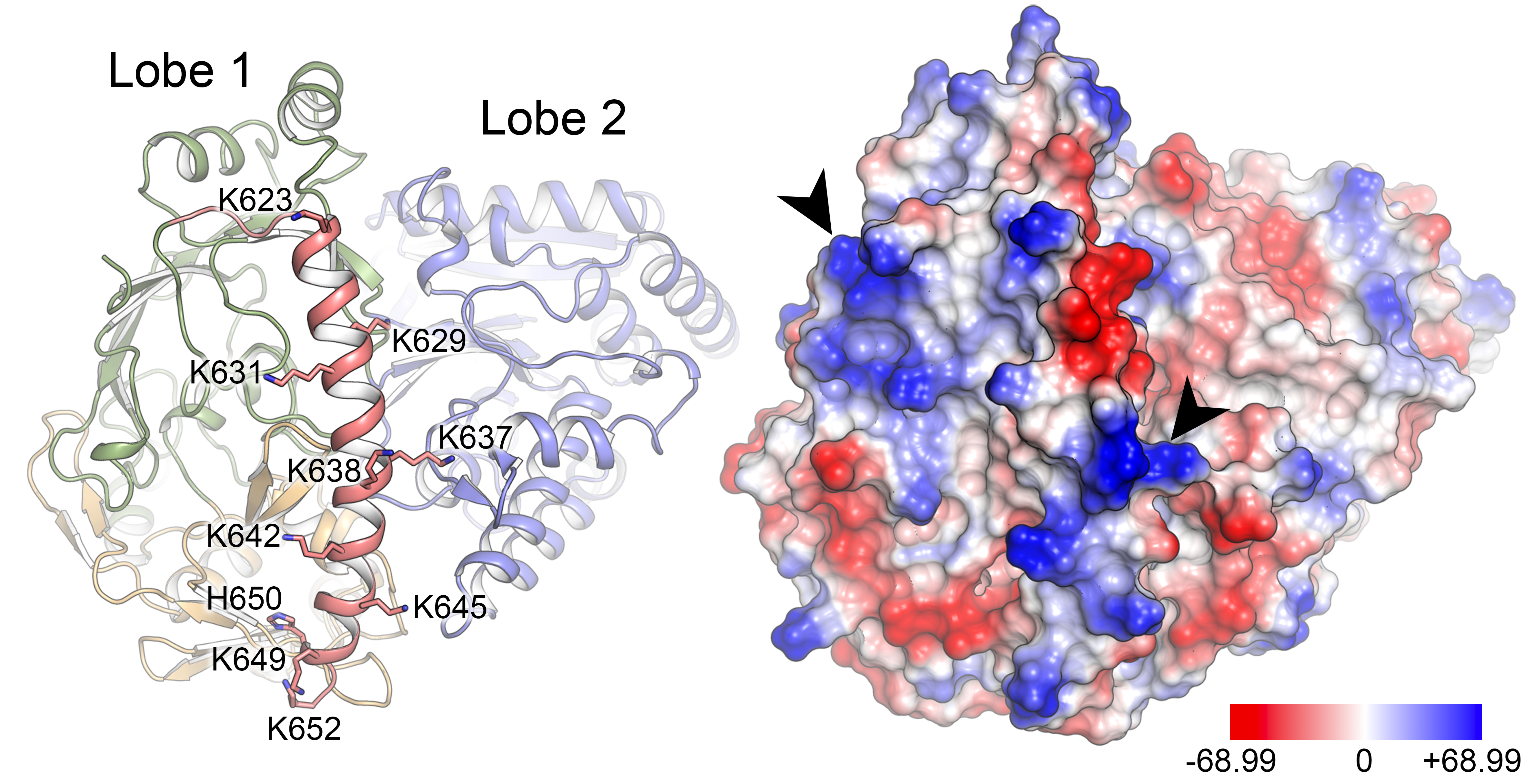

Supplement: S4 Fig — Left panel, cartoon representation of the AliD structure (open conformation) using the same color code as Fig 2B. The focus is on the C-terminal α-helix (α19), where positively-charged residues (mainly lysines) are depicted as capped sticks. Right panel, the electrostatic-potential surface of AliD is shown in the same orientation as the left panel. Relevant basic patches are indicated with black arrows. The color key (blue, positive and red, negative) shows the Poisson-Boltzmann electrostatic-potential surface (color bar range ± 68.99 kT/e). (TIF) [file ppat.1011883.s015.tif]

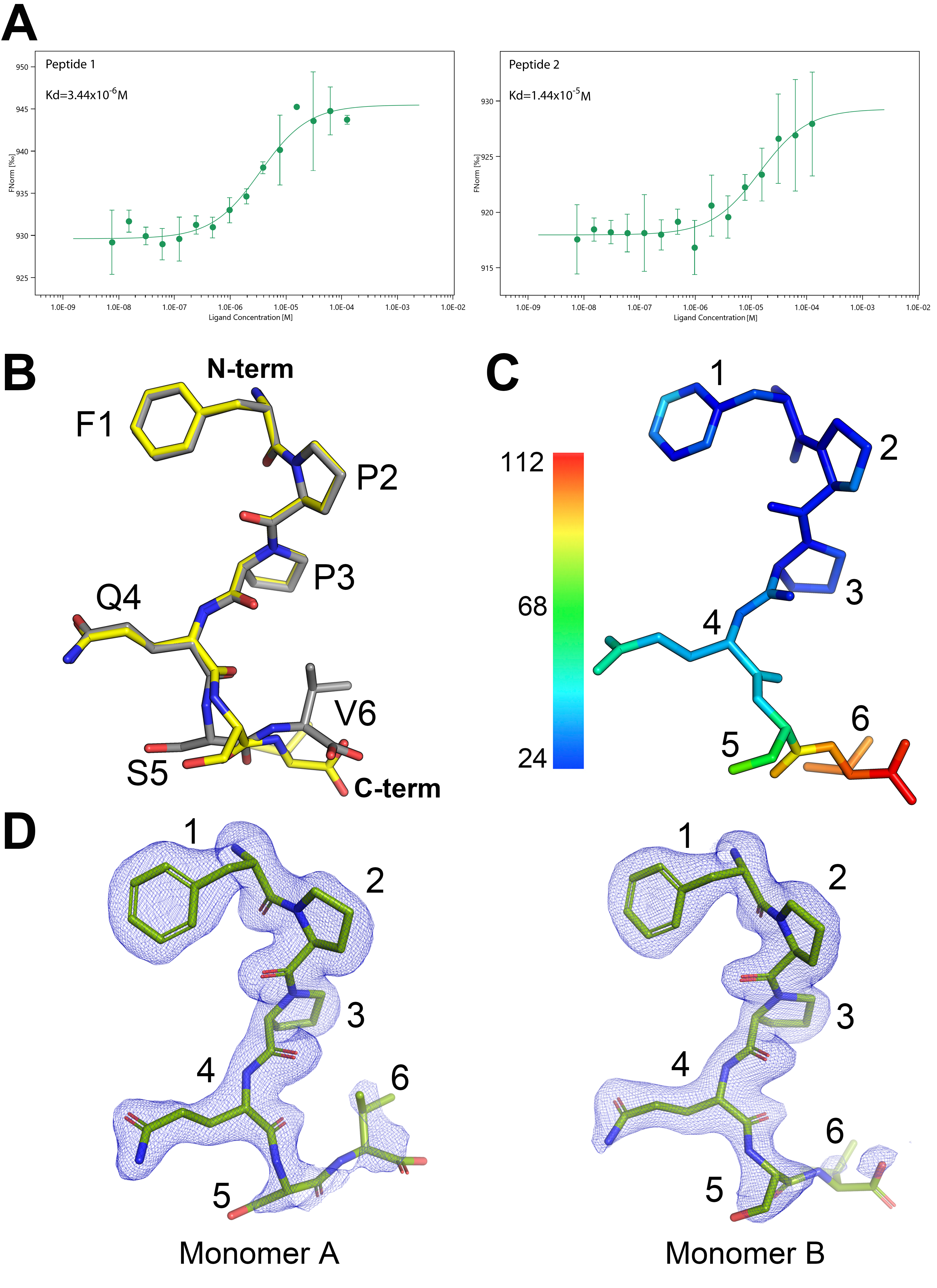

Supplement: S6 Fig — (A) Binding of peptide 1 and 2 to heterologously expressed AliD as analyzed by microscale thermophoresis (MST). The concentration of NHS-RED-labeled AliD was kept constant (20 nM), while the concentration of the non-labeled peptides ranged between 7.63x10^-6^–0.125 mM. Samples were measured using the Monolith NT.115 (NanoTemper Technologies) at 40% LED power and medium MST power at 25° C. The Kd was calculated from three independent measurements, error bars represent the standard deviation. (B) Structural superposition of the two peptide 1 molecules (depicted as capped sticks) bound to AliD monomers A (gray) and B (yellow). The sequence of peptide 1 (FPPQSV) is indicated and numbered. N-term, amino-terminus; C-term, C-terminus. (C) Atomic B factors for peptide 1 as observed in the AliD:peptide 1 complex (monomer B). The Ligand is represented as capped sticks and colored based on the B-factor distribution, ranging from low (blue) to high (red) values. (D) Electron-density map (2mFo-DFc map contoured at 1.0 σ) for each of the two peptide 1 molecules (in green caped sticks) observed in the AliD:peptide 1 complex. The position of each peptide residue is numbered. (TIF) [file ppat.1011883.s017.tif]

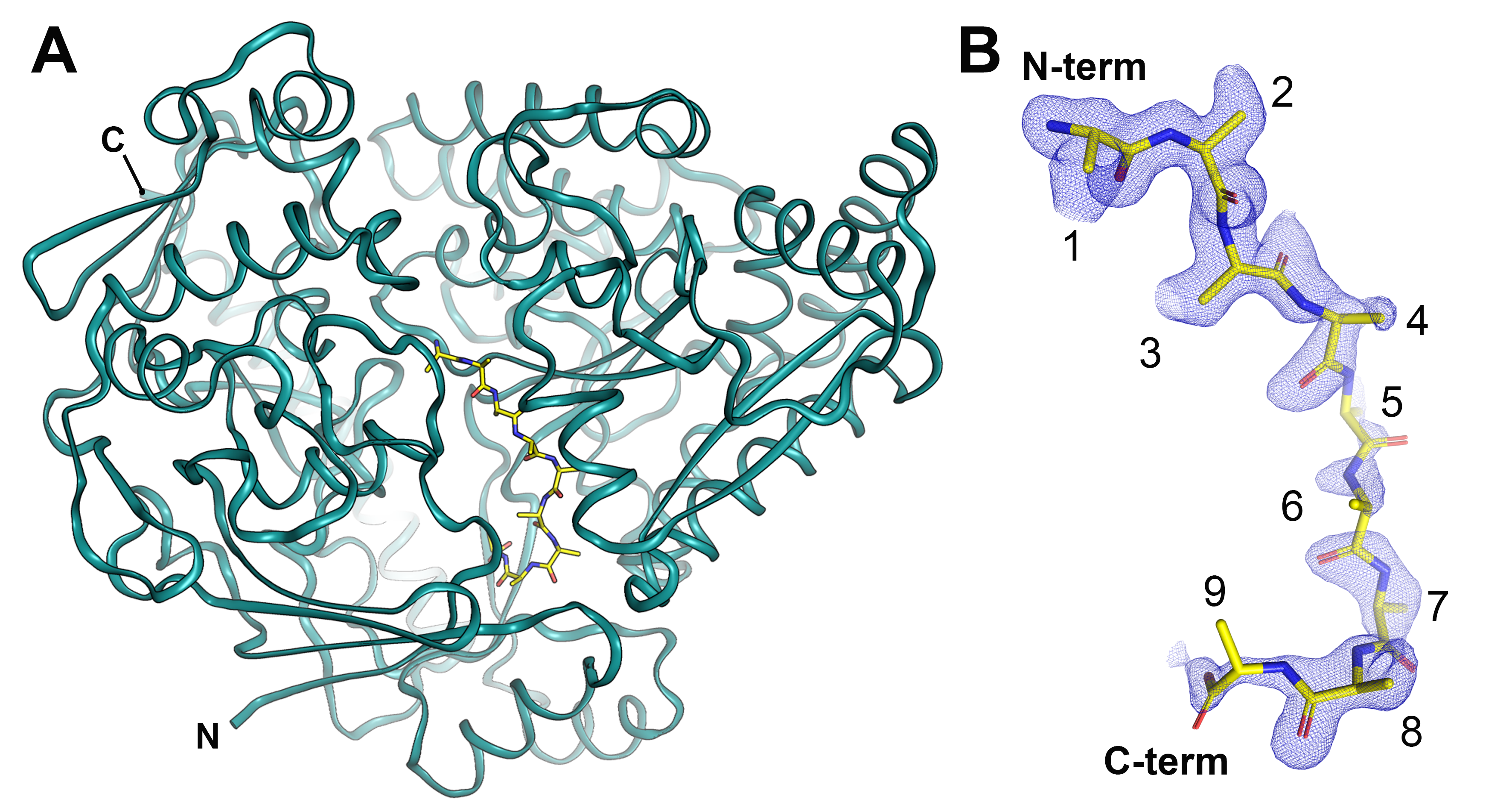

Supplement: S8 Fig — (A) Cartoon representation of AliB structure (colored green) in complex with an unknown oligopeptide(s) from E. coli modeled as poly-Ala (depicted as yellow capped sticks). (B) Electron-density map (2mFo-DFc map contoured at 1.0 σ) of the unknown ligand in which a nine-residues-long poly-alanine backbone has been traced (depicted as yellow caped sticks). The ligand is presented in a similar orientation to that in panel A. The positions for each alanine residue are indicated. N-term, amino-terminus; C-term, C-terminus. (TIF) [file ppat.1011883.s019.tif]

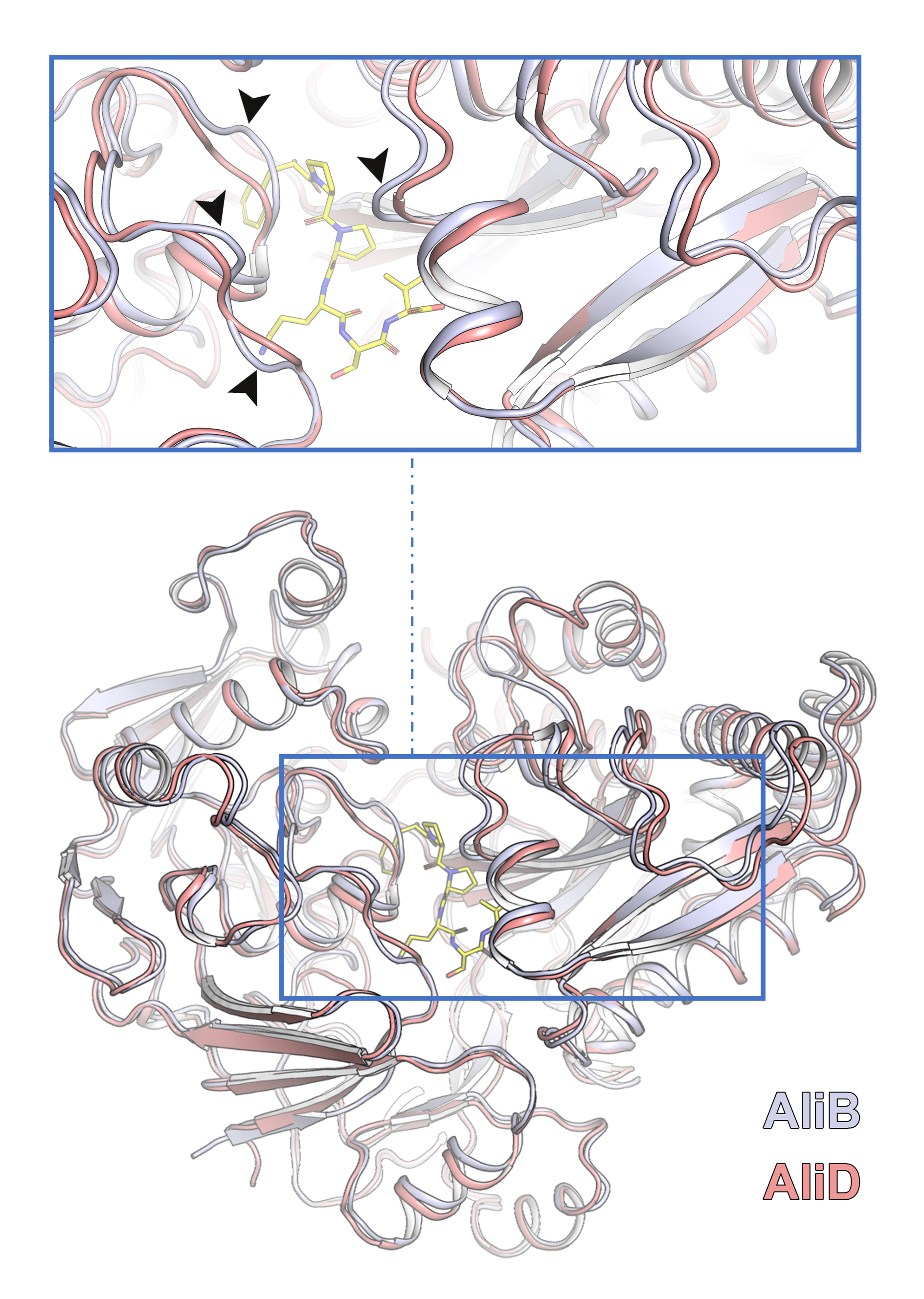

Supplement: S9 Fig — Lower panel, the overall closed conformations of AliD (colored in salmon) and AliB (colored in pale purple) exhibit significant similarity (rmsd 0.88Å for 588 Cα atoms superposition), although AliB’s structure is more tightly closed compared to AliD. Both proteins are depicted in ribbon representation. The upper panel provides a closer view of the region boxed in the lower panel. Differences in the loops shaping the substrate-binding site at positions 1–4 are highlighted with arrows (see main text for details). Peptide 1 (FPPQSV) is depicted as yellow-capped sticks. (TIF) [file ppat.1011883.s020.tif]

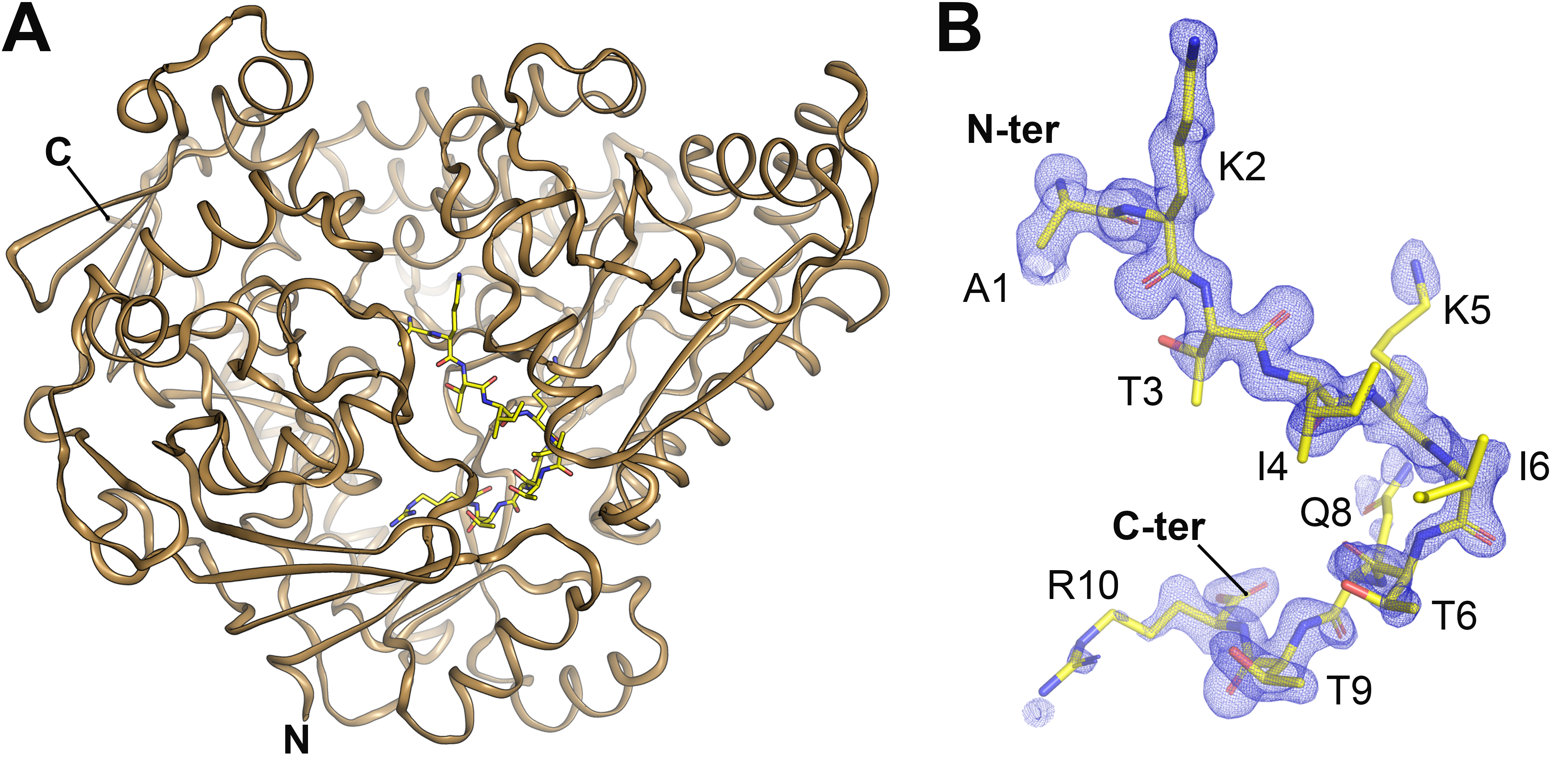

Supplement: S10 Fig — (A) Ribbon representation of AmiA structure in complex with an unknown peptide from E. coli (in yellow caped sticks) that we further refined as peptide 4. (B) Electron-density map (2mFo-DFc map contoured at 1.0 σ) for the 10-residues long ligand has been traced (yellow caped sticks) assuming the sequence of peptide 4 (AKTIKITQTR). Ligand is presented in a similar orientation as the one found on panel A. Positions for each residue are indicated. (TIF) [file ppat.1011883.s021.tif]

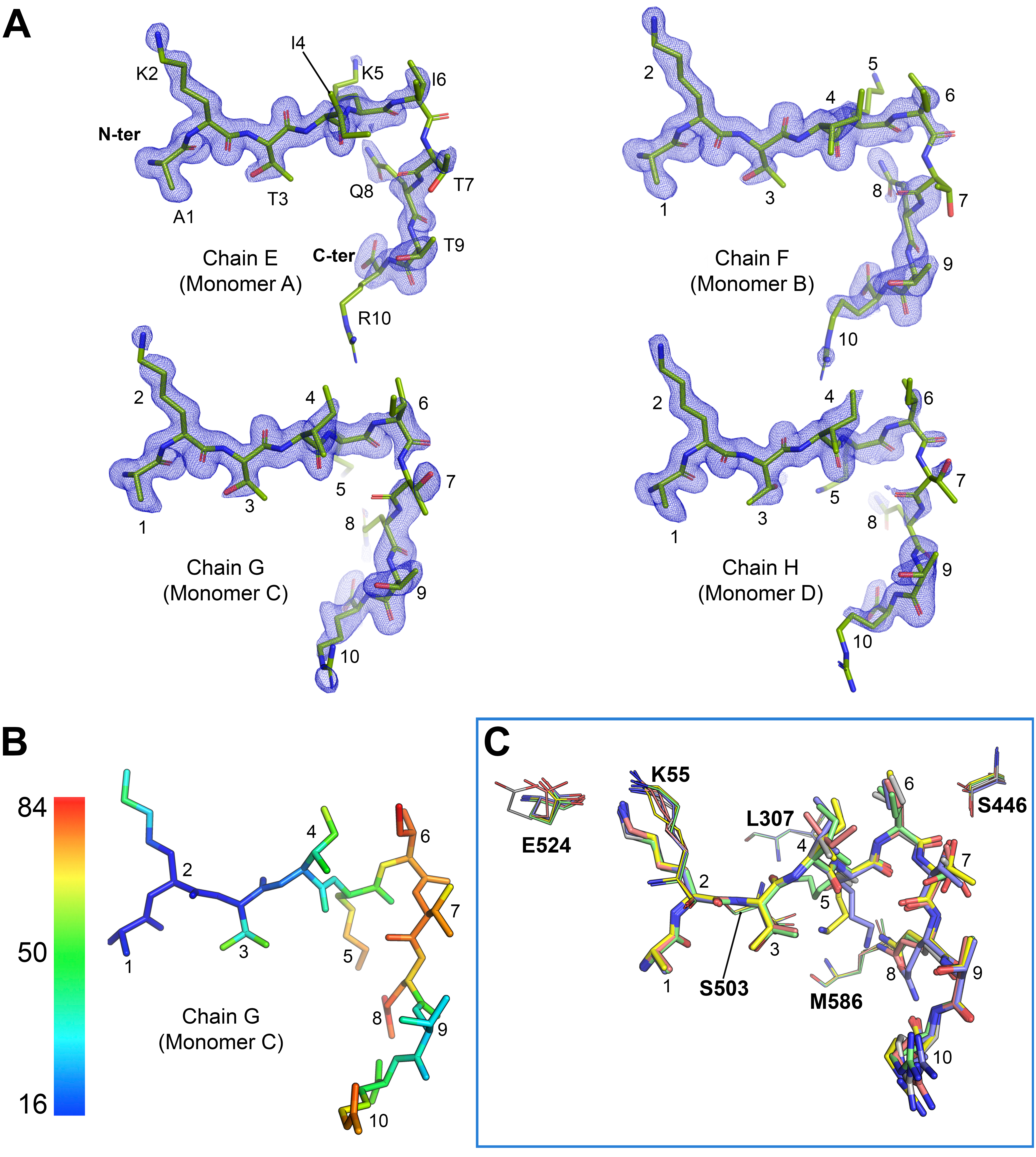

Supplement: S11 Fig — (A) 2mFo-DFc electron-density map for peptide 5 (AKTIKITQTR depicted in green caped sticks) for each of the four monomers (monomers A, B, C and D) as observed in the crystallized AmiA:peptide 5 complex. (B) Atomic B factors for peptide 5 (monomer C). The Ligand is represented as capped sticks and colored according to the B factor distribution, ranging from low (blue) to high (red) values. (C) Structural superposition among the peptide 5 molecules (showed in capped sticks) as observed in the AmiA complexes. Lateral chains from AmiA residues that adopt different conformations are labeled. The peptide sequence is indicated and numbered. N-term, amino-terminus; C-term, Carboxy-terminus. Maps contoured at 1.0 σ. (TIF) [file ppat.1011883.s022.tif]

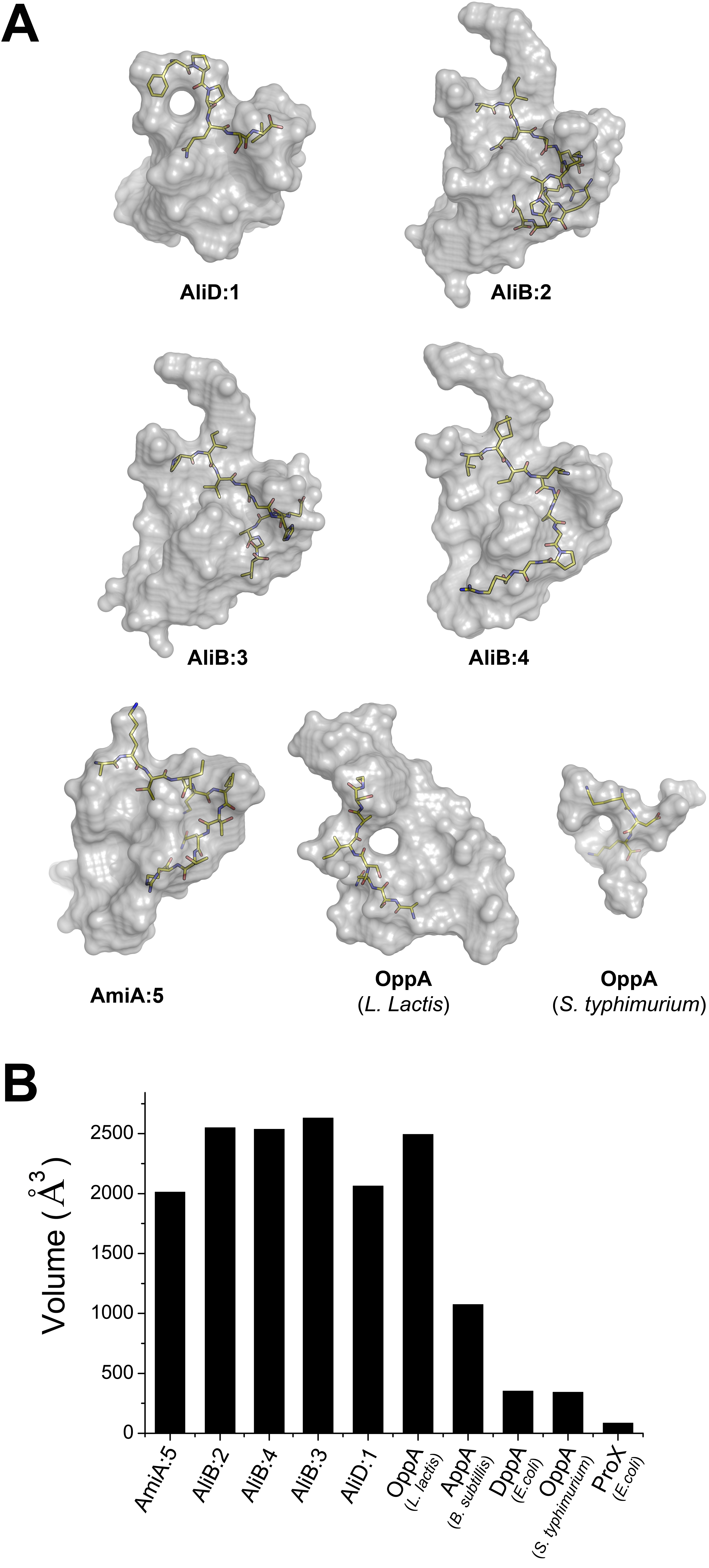

Supplement: S13 Fig — (A) The bound peptides visualized inside the binding cavity of OBPs belonging to the Ami permease reveal the extent of the unoccupied space. The cavity is shown as a gray semi-transparent surface, the peptide is shown in yellow capped stick, and the protein is not shown. AmiA:5, AmiA in complex with peptide 5; AliD:1, AliD in complex with peptide 1; AliB:2, AliB in complex with peptide 2; AmiB:3, AmiB in complex with peptide 3 and AmiB:4, AmiB in complex with peptide 4. (B) Comparison of the binding cavity volumes found on SBPs of the Ami permease, including the cavities of OppA from L. lactis [PDB 3DRF [30]], AppA from B. subtillis (PDB 1XOC [31]), OppA from S. typhimurium [PDB 1B4Z [26]], DppA [PDB 1DPE] and ProX from E. coli [PDB 1SW2 [33]], for comparative purposes. The volumes were calculated with the program POCASA v1.1 [34] using default parameters and a Probe Radius of 1 Å. (TIF) [file ppat.1011883.s024.tif]
