# Supplementary material for: Molecular and structural basis of oligopeptide recognition by the Ami transporter system in pneumococci
Source: PLoS Pathog. 2024 Jun 5;20(6):e1011883. doi: 10.1371/journal.ppat.1011883 (PMC11192437; doi:10.1371/journal.ppat.1011883)
Supplement: S16 Fig — AliD residues interacting with the two permease subunits (AmiC and AmiD) were identified with LigPlot+ v.2.2 [35]. (A) Upper panel, cartoon depiction of AliD (colored orange) is presented in a manner akin to that in Fig 2B (upper panel). AliD residues involved in the interaction with the permease are labeled and depicted in yellow capped sticks, while the semi-transparent representation illustrates the AliD surface. Peptide 1 is visualized as blue spheres. The boxed inset exhibits a surface representation of AliD (orange), highlighting the positions of residues engaging with AmiC (pink) and AmiD (purple). The orientation of AliD corresponds to that in A, upper panel. The middle panel mirrors the representation in A, upper panel, but with a 60° rotation through the indicated axes. The lower panel replicates the view from A, middle panel, incorporating the two permease subunits, AmiC (pink cartoon), and AmiD (soft purple cartoon). ECD, extra-cellular domain. (B) In the upper panel, a table is provided summarizing AliD residues involved in interactions with AmiD. The lower panel presents a table summarizing AliD residues engaged with AmiC. The symbol ’*’ denotes residues that are absolutely conserved across all five OBPs of the Ami system. The symbol ’#’, on the other hand, designates residues with physicochemical properties conserved throughout all five OBPs of the Ami system. (PDF) [file ppat.1011883.s027.pdf]

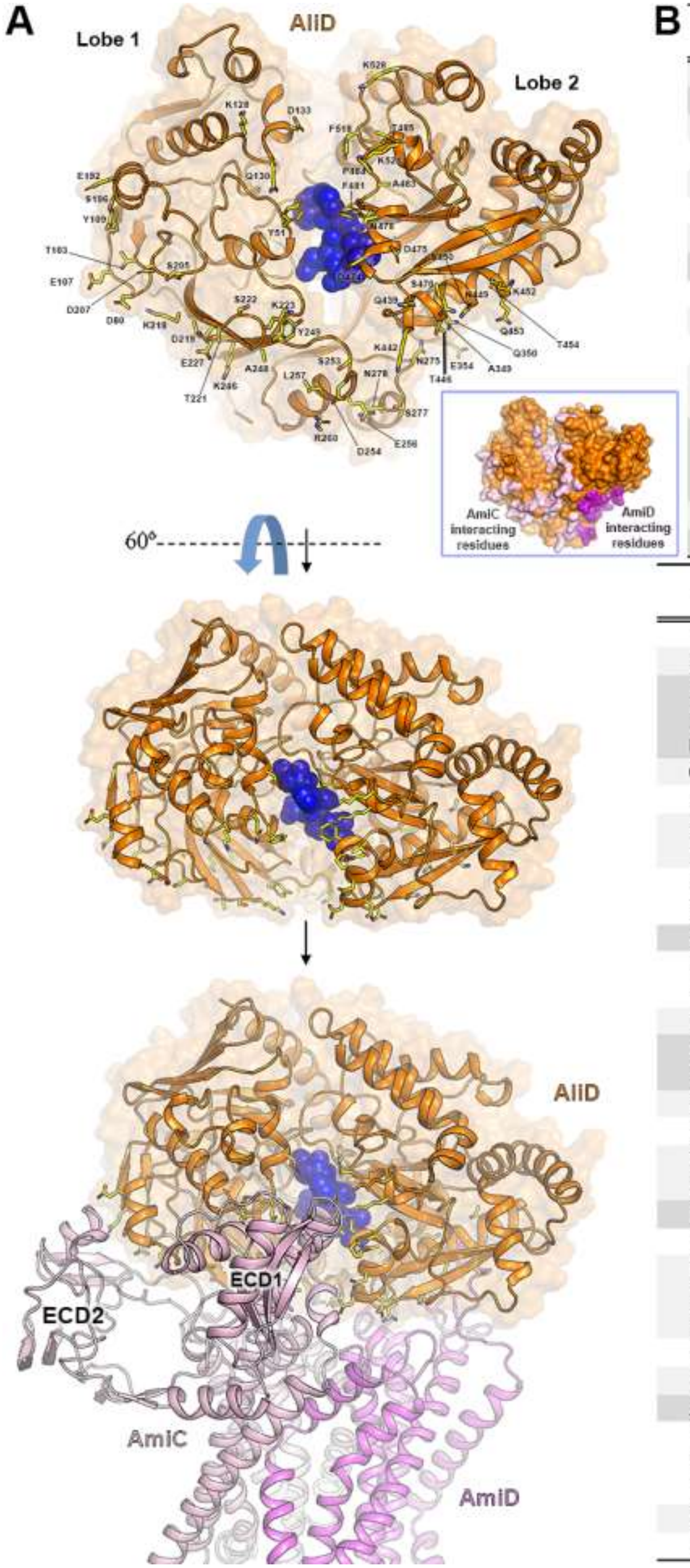

B

AliD	AmiD
S253#	Q268
D254*	Q268
E256#	T272
N275	Y281
S277#	N269
N278	T272
A349#	F77
Q350*	R80
E354	F77
Q439#	I254
K442	D265, Q268
T446#	R80, N95
N449#	V71
S450*	N75
K452*	V71
Q453*	S72, D75
T454#	N75
Q470*	T255

AliD	AmiC
Y51	P41, N42, K45
D105#	T224, S239
E107*	T237, R256
Y109*	D254
K128*	D71
Q130#	N42
D133	K52
E192#	S255
S196#	K248, D254
S205	Y274
D207	S272
K218*	Q223
D219	G222
T221	Q221, P443
S222#	Y208
K223*	K447, D451
E227*	Q223
K246#	P350, I351
A248	P350
Y249#	A441
S253#	Y334
D254*	R337
L257	D347, S348
R260#	D347
D474#	Q39
D475#	K38
N478	K38
F481#	P41, N44, K45
A483*	N44
P484	Y43, A47, R53
T485	A50
F518	T48
K521#	N44, A47
K528	E116
