# Supplementary material for: Molecular and structural basis of oligopeptide recognition by the Ami transporter system in pneumococci
Source: PLoS Pathog. 2024 Jun 5;20(6):e1011883. doi: 10.1371/journal.ppat.1011883 (PMC11192437; doi:10.1371/journal.ppat.1011883)
Supplement: Attachment — Submitted filename: Point-by-point-Comments-22-3-24.docx [file ppat.1011883.s029.docx]

**Reviewer's Responses to Questions**

**Part I – Summary**

Please use this section to discuss strengths/weaknesses of study, novelty/significance, general execution and scholarship.

**Reviewer #1**: Pneumococcus (*Streptococcus pneumoniae*) is a leading cause of pneumonia and is auxotrophic for cysteine, glycine, valine, leucine, isoleucine, arginine, asparagine, histidine, and glutamine. The uptake of diverse oligopeptides enables pneumococcal growth despite auxotrophies and functions as a critical sensor for assessing the composition of the local environment. The uptake of oligopeptides by pneumococci is performed by a specific ABC transporter system known as ‘Ami’. The Ami system includes two transmembrane proteins, AmiC and AmiD, two ATPases, AmiE and AmiF, and five oligopeptide-binding lipoproteins (OBPs), AmiA, AliA, AliB, AliC and AliD. This study presents nine crystal structures of four of the five OBPs within the Ami system, AmiA, AliB, AliC and AliD in two different conformations, closed (with peptide bound) and open. In addition, the authors also performed mass spectrometry analysis to identify the oligopeptides captured by AliB and AmiA during purification. Overall, this study provides valuable information about peptide transport mediated by OBPs within the pneumococcal Ami permease system. However, the manuscript suffers from two aspects and are unsuitable for publication at its current state.

*We appreciate the reviewer's positive evaluation of our work. As described below the revised version of the article has been modified to incorporate suggestions by Reviewer#1.*

**Reviewer #2**: In this manuscript, Alcorlo *et al*, report crystal structures of all the Oligopeptide Binding Proteins (OBPs) of the pneumococcal Ami ABC-transporter system. The authors show the detailed molecular mechanism of how OBPs interacts with oligopeptides. However, the authors do not properly show any biochemical assays to support what their structural observations, and do not report the final refinement validation files of their structures. More importantly, in the manuscript, the authors describe primarily oligopeptide recognition of OBPs, does not provide any experimental evidence for model of oligopeptide transport although the AliD:permease complex predicted by AF, which does not match to their title. Collectively, the manuscript cannot be published until the proper biochemical data are supplemented.

*We have done strong efforts in order to answer all Reviewer’s suggestions. For example, the revised manuscript now contains interaction data showing binding of peptides to the OBP AliD as analyzed by microscale thermophoresis. Also, we tried to obtain structural information about the interaction between the OBP and the transporter without success. Following Reviewer’s suggestion, we have removed the mention to the transport in the Title of the revised manuscript. All the details are described below.*

**Reviewer #3**: This manuscript by Alcorlo *et al*, focuses on the structure of Ami/Ali proteins from *Streptococcus pneumoniae*. These proteins are substrate-binding proteins of ABC transporters that import oligopeptides into the cytoplasm. Because *S. pneumoniae* is an auxotroph for several amino acids, this ABC system is very important during infection by allowing the acquisition of peptides that can serve as sources of these amino acids. This manuscript describes the structure of several oligopeptide-binding Ami/Ali proteins in their apo and substrate-bound states. Some of the peptides bound to Ami/Ali proteins were synthetic peptides already known to bind to these proteins, while others were trapped during protein production in *E. coli*. From these data on substrate specificity, the authors classified the paralogous Ami/Ali proteins into two groups: the AliB-like group and AmiA-like group. Lastly, using modeling, the authors propose a model for the structure and function of this oligopeptide ABC transport system.

Overall, this is a beautiful structural study on a peptide transport system that is important in the physiology of the human pathogen *S. pneumoniae*. The manuscript is very well written, the data are of very high quality, and the figures are highly effective. The model in Fig. 5 is supported by the data and makes sense based on what we already know about other ABC transporters. However, as detailed below, the study needs some additional data to validate the authors’ conclusions on substrate specificity.

*We thank Reviewer #3 for laudatory comments about our work.*

**Part II – Major Issues: Key Experiments Required for Acceptance**

**Reviewer #1**: Key points that need to be addressed are:

**1**. The Ami system has five OBPs for one ABC transporter, AmiA, AliA, AliB, AliC and AliD. Since the authors have nine structures with five of them having clear electron density for the bound peptides. I think the most important question they should address is the peptide preference of each of the OBPs. They should define the peptide binding pocket for each amino acid as P1, P2, P3……, and discuss the amino acid preference of P1, P2, P3……

*We thank the reviewer for her/his suggestion.* *In the revised version of the article, we have incorporated, for each complex, a detailed description of the amino acids constituting each pocket where the side chains of the various peptides utilized in this study are accommodated. This information is documented in the form of new supplementary tables (****S3-S7 Tables****), to which the main text refers as it delineates the different complexes.*

**2**. The authors performed mass spectrometry analysis of the oligopeptides captured by AliB and AmiA during purification. They observed distinct tendency to bind specific peptides. Given that they have the structures of AliB and AmiA, obviously they should map the distinct tendency back to the structures and provide a structural view of tendency.

*To address this question raised by the reviewer in the revised version we have described in more detail how the structural accommodation of the peptides detected by mass spectrometry can be explained considering the physicochemical properties of the OBP pockets, with a focus on the first three-four positions of each peptide.*

**Reviewer #2**:

(**1**) The authors must supply the validation reports for all the reported structures to verify deposition and to accurately judge the models. This manuscript cannot be accepted for publication without proper validation of the structures.

*We wholeheartedly agree with the reviewer. We deeply regret not having submitted the validation reports at the time of article submission. In the submission of the revised version of the manuscript, we have included the validation reports for all the nine crystallographic structures here reported.*

(**2**) The authors describe the domain-swapped dimer architecture of AliC, distinct from other OBPs. I think this point is important, however, the authors do not further elaborate or discuss it further.

*We thank Reviewer#2 for her/his interesting suggestion. We have omitted this information in the original version to match the size limitations of the journal. Following the reviewer's instructions, we have now proceeded to integrate further information on the domain-swapping observed in AliC.*

Whether this conformation is physiological or is caused by molecule packing during crystallization should be answered.

*To address the question posed by the reviewer, we have investigated the self-association state of AliC in solution through analytical ultracentrifugation experiments. Utilizing sedimentation velocity assays within the range of 4.1-113 µM, we observed that approximately 30% of the protein exists in a dimeric state, regardless of the concentration value analyzed. As a control, we also included AliD which behaved as a monomeric species, as can be seen in the figure below, which shows the c(S) distribution for both proteins at the particular concentration of 4.1 µM. While the biological implications would require further investigation (the OBPs remain attached to the membrane), this reflects the genuine behavior of the AliC protein in solution and rules out the possibility that domain exchange is attributable to crystal packing effects.*

*The peaks at ~4.7 S and ~7.0 S are consistent with the theoretical masses of the monomer (70.37 kDa) and globular dimer (140.74 kDa), respectively, of the protein.*

*This information has been included in the revised version, and the AUC data included as a new panel in the* ***S5 Fig****.*

(**3**) The authors claim that the C-terminal α-helix (α19 in AliD) as the distinctive hallmark of all the Ali proteins, contributes to the stabilization of Lobe1 on the membrane (Line 231-233). The authors should supply proper biochemical assays, *e.g.* liposome co-sedimentation using OBP mutants lack of this α-helix.

*We appreciate the reviewer's observation. The message we aim to convey here is that this C-terminal alpha helix is a structural feature unique to pneumococcal OBPs. This helix plays a structural role by masking numerous hydrophobic residues in the Lobe 1. Besides, it presents, overall, an exposed basic character. We thus hypothesized the putative implication of this helix in orienting the protein on the membrane, favoring interaction with its substrate, rather than asserting a strong protein-membrane interaction through this α-helix (real attachment with the membrane is produced by lipidation of Cys23 at the beginning of the unfolded N-terminal region).*

*In alignment with this rationale, we have revised this section of the manuscript to soften this speculative aspect. In the same vein, panel C in S4 Fig. has been also removed in the revised version.*

(**4**) In the manuscript, although the authors report several complex structures of OBPs with oligopeptides and reveal the key residues involved in oligopeptide-binding structurally, it is still lack of proper biochemical verification. The authors should provide biochemical-binding assays (*e.g.* ITC or MST etc.) to confirm their structural observations.

*All the oligopeptides in complex with OBPs presented in our work were previously described to bind in vivo to these specific OBPs (Hathaway et al Open Biol (2014), 4; Nasher et al Front Microbiol (2018), 8, 318591). We apologize if this information was not clearly transmitted in the original version.*

*Following the Reviewer’s comment, we have performed interaction studies using AliD as prototype OBP. Peptide-binding to AliD was investigated by microscale thermophoresis (MST) and the data are now included in the revised version of our manuscript. A new panel has been included in* ***S6 Fig*** *with the MST data.*

(**5**) In the AF model, how AmiC or AmiD recognizes AliD in closed or open state. The authors should provide the critical sites involved in interaction of AliD with AmiC/AmiD and corresponding biochemical evidence to support their model.

*Following the reviewer's advice, in the revised version we have proceeded to elaborate on the interaction sites of AliD in its closed conformation with the permease. We have systematically dissected all interactions occurring between AliD and the two permease subunits (AmiC and AmiD). The outcomes of this analysis have been compiled in a novel supplementary figure (****S16 Fig****).*

*As can be seen, it is a complex interface that involves many residues and interactions of diverse nature. These properties make this system challenging to approach through directed mutagenesis, as it raises the dilemma of how many mutations would be needed to achieve a drastic phenotype.*

*Following Reviewer#2 suggestion we have unsuccessfully tried to validate the AF model. According to AF´s predictions, in the ABC transporter only AmiC presents a folded extracellular domain (AmiC_ECD_, residues 34-124, see attached figure) that would interact with the two lobes of the OBPs, when they adopt a closed conformation. We thus attempted to produce, purify and crystallize this domain (alone and in complex with AliD) through two approaches: 1) we produced it with a histidine tag at the N-terminal end. However, the production was extremely low, and we could not achieve the protein levels necessary for structural characterization. 2) we produced the mentioned domain fused to SUMO. In this case, we obtained the fusion protein with a good yield. However, the Ulp1 protease could not cleave the fusion, suggesting that the domain might not be correctly structured. As Ulp1 was unable to cleave the fusion, we resorted to using ammonium sulfate (AS) for a selective precipitation of the protein and contaminants, allowing us to attain the protein with the highest achievable degree of purity. Nevertheless, we conducted crystallization assays with the fusion but were unsuccessful in obtaining crystals. The workflow used is summarized in the figure below:*

*We will continue our structural characterization of the system by other ways like cryo-EM, but this objective is beyond the present manuscript. We hope the Reviewer will appreciate the efforts we have done in order to answer her/his suggestion.*

Whether the recognition of other OBPs by AmiC/AmiD is conserved?

*The recognition of the five different OBPs (AmiA, AliA, AliB, AliC, and AliD) by the permease is conserved in the sense that all the OBPs are predicted to be placed in the same way onto the permease and involving the same regions in both the OBPs and the permease. Obviously, due to the large extension of the interfaces involved (****S16A Fig****) and the sequence variation among the OBPs (sequence identity 50-58%), not all the interactions should be conserved. In the analysis provided in the new S16 Fig it has been detailed all the interactions (as predicted in the AF2 model) between the AliD and the transporter (AmiC, AmiD). Interestingly most of the residues involved in the interaction are conserved as indicated in the table of* ***S16B Fig****.*

*We have detailed this information in the revised version of the manuscript.*

In addition, it is better that the panel A of Figure 5 should be shifted to the last panel.

*Done as requested*

(**6**) S6-10 Figs: The authors show 2Fo-Fc maps with the peptide. I think this is a 2mFo-DFc?

*The reviewer is right and we appreciate their observation. This has been corrected in the revised version.*

The authors need to also show the mFo-DFc maps of peptide as well.

*To fulfill the reviewer's request, we have produced a novel supplementary figure (****S7 Fig****) illustrating the mFo-DFc maps (contoured at 2.5 σ) corresponding to the ligands within all presented complexes. Additionally, within the same figure, we have incorporated the 2mFo-DFc maps (contoured at 1.0 σ) for each peptide, as observed after refinement.*

Additionally, a contour of 0.8 sigma is a bit low for displaying ligand density, especially given the high resolution, it is unclear if the ligand density is due to model bias. Was the ligand not well bound?

*The various peptides are securely bound in the presented structures. Electron density is particularly well-detected in the initial positions of the peptide, which are the most strongly stabilized through many polar and van der Waals interactions. However, depending on the specific peptide, there are regions, particularly at the C-term region, where the density is not so nicely defined, indicating that these portions of the substrate are not tightly bound to the OBP and in agreement with both B-factors (****S6C*** *and* ***S11B Figs****) MST data (****S6A Fig****). The contour level was lowered to 0.8 sigma to emphasize the density of these weakly interacting regions. However, in response to the reviewer's concerns, we have proceeded to re-elaborate all figures depicting ligand densities, in this instance utilizing a contour of 1.0 sigma.*

**Reviewer #3**:

**1**) The study lacks validation of the conclusions on substrate specificity. For example, the authors concluded that the first 3 residues of substrate peptides share common features that determine specificity for AliB. They also know how those features interact with the substrate-binding pocket. I think that this study would be strengthened if, based on their model, the authors generated a few protein variants predicted to be defective in substrate binding as well as a few artificial substrates that would be predicted to bind. Binding could be tested using standard biochemical binding assays (no structures required). Ideally, this could be done with a member of each the AliB-like and AmiA-like groups.

*The comment raised by Reviewer #3 points to one of the most relevant results in the manuscript. Our unanticipated discovery of hundreds of different peptides bound to AliB and AmiA trapped during the purification process, reveals that not a strong substrate specificity is required in the pneumococcal OBPs. This agrees with our MST measures showing Kd in the μM range. The analysis of the huge number of peptides experimentally detected by mass spectrometry (****S12 Fig****.) that are attached to the same OBP (AliB or AmiA respectively) reveals that, even at the N-term region of the oligopeptide in which our crystal structures show strong network of interactions, there is not a strict sequence requirement as seen in the sequence logo (****S12B Fig.****). Three-dimensional structures of the different complex (three of them with the same OBP, AliB) provided a nice explanation on how oligopeptides can be recognized even without a strong sequence conservation: the N-terminal residues of the substrate must form an anti-parallel β-strand interaction with β20 of Lobe 2 (encompassing the first three positions for the AliB group and four positions for the AmiA group), this strict structural requirement can be fulfilled by different combination of residues, and the affinity modulated by the nature of the oligopeptide-side chains that are differently accommodated depending on the electrostatic nature of the here identified binding-pockets (summarized in* ***Fig. 4D****). After the fifth position, the oligopeptide is not strongly recognized, even structurally as observed for peptides* ***2*** *or* ***3*** *that present radically different 3D structures (α-helix vs coiled coil) (****Fig. 3****) thanks to the large binding-site cavity of these pneumococcal OBPs (the largest among OBPs,* ***S13 Fig****). Thus, our results change the paradigm of substrate recognition by OBPs that, at least in the case of S. pneumoniae (as nicely pointed by Reviewer #3 in her/his following comment) could have profound biological implications.*

*This conclusion has been further elaborated in the revised version of the manuscript.*

**2**) The authors identified peptides that were bound to purified Ami/Ai proteins that were produced in *E. coli*. Then, they determined the proteins that the peptides were derived from and discussed the various functions of these proteins (page 20). They noted that many of these proteins are ribosomal proteins or proteins associated with ribosomes. They also highlighted metabolic proteins and proteins involved in transcription, DNA replication or macromolecule transport. I do not understand the significance of analyzing the type of functions of these proteins (Fig. S11). The significance would have to be explained. To me, it is likely that there is an enrichment of ribosome-derived peptides because ribosomes are very abundant and large. Are most of the peptides derived from abundant proteins?

*We agree with Reviewer#3 about the main conclusion of this analysis. The finding of such a large number of oligopeptides trapped during purification process of AmiA and AliB was completely unexpected and never seen in other OBPs. In order to rationalize this finding, we analyzed the nature of the E. coli proteins as shown in (****S12 Fig****.) to see if there were any specific preference. We concluded as Reviewer#3 that this must be related with the abundance of these proteins in E. coli. This aspect has been further stressed in the revised version and the paragraph with the analysis of the functions of the proteins shortened.*

*Another relevant conclusion derived from this study is that rather than specific, these two proteins present a large promiscuity in their substrates and pointing to a new view on the mechanism: the oligopeptides are mainly recognized by the initial residues capable to pack as a beta-strand with the beta 20 from Lobe 2, and then there is a great variability that can be accommodated in the huge internal cavity (among the largest in OBPs) formed between the two lobes. This also explain why peptide* ***2*** *(a reported substrate of AliB) shows binding by MST to AliD (****S6A Fig****).*

It is yet to be determined if Ami proteins evolved to recognize peptides derived from abundant and conserved proteins such as ribosomal proteins. Moreover, the peptides that were trapped are derived from cytoplasmic proteins because the Ami/Ali proteins were produced in the cytoplasm of *E. coli*. Naturally, this is not the cellular compartment where Ami/Ali protein’s function. Therefore, for the type of proteins (peptides) described by the authors to be relevant, they would have to be released from the cytoplasm likely through lysis. Given that *S. pneumoniae* undergoes autolysis, and if these peptides are also found in the cytoplasm of *S. pneumoniae*, is this transport system relevant in the context of the autolysis program of this bacterium? Currently, the Discussion section lacks an insightful discussion on the physiological role of this system. It might be worth considering this point if applicable.

*We thank the Reviewer#3 by this extremely interesting observation. All the autolysins in S. pneumoniae are modular proteins containing a catalytic module exhibiting lysozyme (e.g. LytC), glucosaminidase (e.g. LytB), amidase (e.g. LytA), or endopeptidase (e.g. CbpD) activities, and a cell-wall binding domain that specifically binds the choline moieties present in the pneumococcal wall-teichoic and lipo-teichoic acids. These autolysins are involved in cell division (LytB) and in fratricide (LytA, LytC and CbpD). Particularly, fratricide mechanism is trigger at the end of the regulon of competence resulting in lysis of non-competent pneumococcal siblings. While the obvious DNA incorporation by pneumococcus in this competent state has been studied, there is no information, to our knowledge, about the oligopeptide incorporation associated to pneumococcal competence. Thus, Reviewer’s idea is particularly stimulating, even more considering that some pneumococcal OBPs (AliC, AliD), are only present in non-encapsulated pneumococci (NESp). We thank Reviewer#3 for her/his suggestion. This hypothesis has now been included in the Discussion section of the revised version.*

**Part III – Minor Issues: Editorial and Data Presentation Modifications**
Please use this section for editorial suggestions as well as relatively minor modifications of existing data that would enhance clarity.

**Reviewer #1**: (No Response)

**Reviewer #2**: Note to authors: there are multiple errors of typo. Below is only an example for the authors’ reference.

*We greatly appreciate the reviewer for their diligent correction efforts. Following their guidance, we have conducted a comprehensive revision of the entire manuscript. We anticipate that, as a result of this process, the numerous typographical errors have been thoroughly rectified.*

(1) Line 411: the color of parentheses should be black.

*Done.*

(2) Line 547: the color of “AF” should be black.

*Done.*

(3) Line 740: the underline of unit “℃” should be removed.

*Done.*

(4) Figure 4D & S6-10 Figs.: Residue labeling should be consistent with others.

*Done.*

(5) Line 1159: “Figure S11.” should be “S11 Fig.”?

*Done.*

(6) Figure S11A: the X-axis labeling “Lenght” should be “Length” in x-axis.

*Done.*

**Reviewer #3**:

1) Line 179: "652); were..." should have a comma, not a semicolon.

*Done.*

2) Table S1: It would be better to use protein names as titles of columns instead of numbers.

*Done.*

3) In Materials and Methods: Please clarify that protein variants lacking their respective signal sequences were produced in the cytoplasm of *E. coli*.

*Done.*
